# The Modality Preferred by Population of Health Services Provided During COVID-19 and Assessment of Satisfaction Among Adults in Kingdom of Saudi Arabia

**DOI:** 10.7759/cureus.16190

**Published:** 2021-07-05

**Authors:** Amer H Assiri, Naif A Mahnashi, Nora F Alkhttabi, Khalid M Alqahtani, Mushary S Alqahtani, Meshal A Alhajri

**Affiliations:** 1 Internal Medicine, King Khalid University, Abha, SAU; 2 Medicine, Najran University, Najran, SAU; 3 Medicine, King Khalid University, Abha, SAU

**Keywords:** covid-19, pandemic, health care, modalities, telemedicine, satisfaction, perception

## Abstract

Background

During the COVID-19 pandemic, there was a great transition in the modalities of health care services, such as the telemedicine landscape, with some speed. Because of the lack of vaccines or effective therapies, social distancing and quarantine were the only widely accessible precautions, creating a compelling reason for alternatives for in-person care. Many countries applied teleconsultation or provided online applications during the COVID-19 pandemic. However, it is not currently known whether this available service has satisfied the patients' needs during the COVID-19 pandemic.

Aim

To detect the preferred modality of health services by the Saudi population during COVID-19 and to assess the satisfaction with respect to the provided health services in the Aseer region of Saudi Arabia.

Methodology

A descriptive cross-sectional approach was used targeting all accessible populations in Saudi Arabia. Data were collected from participants using an electronic pre-structured questionnaire. The tool covered participants' socio-demographic data, participants’ medical and family history, COVID-19 infection, health problems during the pandemic, received services, modalities of available services, and their satisfaction regarding the provided health service.

Results

A total of 2102 participants completed the study questionnaire. Exactly 773 (36.8%) respondents had a health problem during the COVID-19 pandemic. The most-reported modality of health services used was calling the 937 number, which is call center of the Ministry of Health (34.7%), followed by using health applications to get an appointment (33.9%). Also, 447 (88.7%) participants reported that the provided medical services were helpful. Regarding their satisfaction, 156 (31%) were just satisfied with the provided services and 280 (55.6%) were highly satisfied.

Conclusions

In conclusion, the current study showed a high level of public satisfaction regarding different modalities of health services provided during the COVID-19 pandemic in Saudi Arabia. This satisfaction was moderately high among telehealth users and those who had hospital visits for health care.

## Introduction

In December 2019 and the beginning of 2020, the world was challenged by a speedily spreading epidemic of coronavirus disease 2019 (COVID-19) caused by severe acute respiratory syndrome coronavirus 2 (SARS-CoV-2) which later was classified by WHO as a pandemic [1]. The outbreak started in Wuhan, Hubei province, China, in December 2019. The World Health Organization classified the disease as a Public Health Emergency of International Concern on 30 January 2020 and recognized it as a pandemic on 11 March 2020 [2-3].

In Saudi Arabia, the government applied prompt measures to prevent and limit the spread of COVID-19. Saudi Arabia implemented policies of availability of personal protective equipment, stay-at-home, social distancing, and quality hospital care to the people.

Sudden situations like the COVID-19 pandemic lay a massive burden on health care service providers to rearrange the hospital organization and policies to minimize or even prevent the spread of fatal infections and certify smooth functioning of health care delivery [4]. During the pandemic, many alternative modalities for health care provision were initiated. It was a suitable method to provide low-risk urgent care for non-COVID-19 cases, including clinical assessment, follow-up of certain chronic medical conditions, and follow-up of patients after hospitalization [5]. Many countries applied teleconsultation or provided online applications during the COVID-19 pandemic [6-9]. However, it is not currently known whether these services have satisfied the patients' needs during the COVID-19 pandemic so far [10]. The current study aimed to detect the preferred modality of health services by the Saudi population during COVID-19 and to assess patient satisfaction with the provided health services.

## Materials and methods

Methodology

A descriptive cross-sectional survey was applied levellingall available populations in Saudi Arabia. Participants above 18 years of age living in Saudi Arabia were invited to participate in the survey. Data were collected from participants using a pre-structured electronic questionnaire. The researchers created the questionnaire after intensive literature review and expert consultation. The tool was reviewed using a panel of three experts from the College of Medicine at King Khalid University to check its applicability and its content validity. Tool reliability was assessed using a pilot study of 25 participants with a reliability coefficient (Cronbach’s α) of 0.71. The questionnaire included the following data: participants' socio-demographic data like age, education, and residence region. The second section included information regarding having illness during the COVID-19 pandemic and the modality of received health care. The last section covered items of participants' perception and satisfaction regarding the provided health service. The questionnaire was uploaded online using social media platforms by researchers and their friends and all eligible persons were invited to fill it after explaining the purpose and conforming to their data confidentiality.

Data analysis

After data was collected, it was modified, coded, and entered into statistical software IBM SPSS version 22(IBM Corp., Armonk, USA). All statistical analysis was done using two-tailed tests. P-value less than 0.05 was considered to be statistically significant. Descriptive analysis based on frequency and percent distribution was done for all variables including participants’ residence, age, history of illness during the COVID-19 pandemic, type of services received, and their satisfaction level. Cross tabulation was used to test for the distribution of participants' satisfaction level regarding health services provided during the COVID-19 pandemic by their factors. Pearson chi-square test was used for testing significant associations at cross-tabulation.

## Results

A total of 3000 eligible persons received the study survey; 2102 people completed the questionnaire with a response rate of 70.1%; 768 (36.5%) were from the Southern region, 477 (22.7%) from the Western region, 410 (19.5%) from the Central region, 257 (12.2%) from the Northern region; and 190 (9%) from the Eastern region. Participants' ages ranged from 18 to 60 years with a mean age of 26.8 ± 11.4 years; 75.5% of the participants were university graduates and 21.3% had a secondary level of education (Table 1).

**Table 1:** Personal data of study participants

Personal data	No	%
Region		
Central region	410	19.5%
Eastern region	190	9.0%
North region	257	12.2%
Western region	477	22.7%
Southern region	768	36.5%
Age in years		
18-22	760	36.2%
23-30	682	32.4%
31-40	306	14.6%
41+	354	16.8%
Educational level		
Below secondary	67	3.2%
Secondary	447	21.3%
University / above	1588	75.5%

Table 2 shows the modality of health services provided during COVID-19 preferred by the population. A total of 773 (36.8%) respondents had a health problem during the COVID-19 pandemic. The most-reported modality of health services provided was calling 937 number (34.7%), followed by using health applications to get an appointment (33.9%), visiting primary health care centers (27.8%), visiting private hospitals (27.4%), while only 4.4% received health care the government hospitals, 1.6% had home care, and three persons (006%) made an ER visit. 

**Table 2:** Modality of health services provided during covid-19 preferred by the population

Medical care data	No	%
Had any health problem during COVID-19 pandemic?		
Yes	773	36.8%
No	1329	63.2%
If yes did you seek medical help? (n=773)		
Yes	504	65.2%
No	269	34.8%
How did you receive the health care (n=773)		
Calling 937 number	175	34.7%
Health application for appointment	171	33.9%
Primary health care	140	27.8%
Private hospital	138	27.4%
Hospital visit	22	4.4%
Home care	8	1.6%
ER visit	3	.6%

Table 3 illustrates participants' satisfaction with the health services provided during the pandemic. A total of 447 (88.7%) participants reported that the provided medical services were helpful. Regarding their satisfaction, 156 (31%) were just satisfied with the provided services and 280 (55.6%) were highly satisfied.

**Table 3:** Participants' satisfaction with health services provided during COVID-19 pandemic

Satisfaction	No	%
Were the medical services helpful? (n=773)		
Yes	447	88.7%
No	57	11.3%
Satisfaction regarding provided medical service (n=773)		
Very dissatisfied	18	3.6%
Dissatisfied	24	4.8%
Neutral	26	5.2%
Satisfied	156	31.0%
Very satisfied	280	55.6%

Table 4 clarifies the distribution of public satisfaction levels regarding health services provided during the COVID-19 pandemic by their factors. About 98.4% of the participants from the northern region were satisfied with the provided health service compared to 97.1% from the eastern region and 81.3% from the western region (P=0.004). Also, 93.3% of the participants aged 18-22 years were satisfied compared to 80% of those aged 41 years or more (P=0.008). Further, 95.5% of participants who received health care in hospitals were satisfied compared to 82.9% of those who had the service at primary health care centers (PHCCs) and 37.5% of those who stayed at home (P=0.003). Higher satisfaction was also reported among those who think that the medical services were helpful than others who did not (92.8% vs. 36.8%, respectively; P=0.001).

**Table 4:** Distribution of public satisfaction level regarding health services provided during the COVID-19 pandemic by their factors P: Pearson X2 test *P<0.05 (significant)

Factors	Satisfaction regarding provided medical service				p-value
	Unsatisfied/ neutral		Satisfied		
	No	%	No	%	
Region					.004*
Central region	13	12.3%	93	87.7%	
Eastern region	1	2.9%	33	97.1%	
North region	1	1.6%	61	98.4%	
Western region	24	18.8%	104	81.3%	
Southern region	29	16.7%	145	83.3%	
Age in years					.008*
18-22	11	6.7%	152	93.3%	
23-30	34	18.0%	155	82.0%	
31-40	9	11.4%	70	88.6%	
41+	14	19.2%	59	80.8%	
Educational level					.396
Below secondary	3	23.1%	10	76.9%	
Secondary	11	10.6%	93	89.4%	
University / above	54	14.0%	333	86.0%	
How did you receive the health care					.003*
Home care	5	62.5%	3	37.5%	
Health application for appointment	22	12.9%	149	87.1%	
Calling 937 number	25	14.3%	150	85.7%	
Primary health care	24	17.1%	116	82.9%	
ER visit	0	0.0%	3	100.0%	
Private hospital	18	13.0%	120	87.0%	
Hospital visit	1	4.5%	21	95.5%	
Were the medical services helpful?					.001*
Yes	32	7.2%	415	92.8%	
No	36	63.2%	21	36.8%	

## Discussion

The current study aimed to define the modality of health services preferred by the population during COVID-19. It also aimed to assess their satisfaction regarding the provided health services. COVID-19 was highly and widely propagating across the whole world and in Saudi Arabia by March 2020 [11-12]. This was associated with high public fear of getting the infection when they go to hospitals or any health care facility for treatment, medical advice, and follow-up. This motivated health care authorities to update technologies that enable them to continue living in their house and be more informed and engaged in their own health and minimizing exposure to health care facilities [13-15].

Saudi Arabia faced the challenge of the pandemic by implementing disease control measures and efforts to satisfy the community’s needs and challenges in a very short time [16]. About 30.26 million people in Saudi Arabia use the internet and 96% of the population depends on smartphones [17], and most of the population now has access to smartphones, laptops, PCs, and tablets; consequently, digital service delivery became easier than in the past and has aided the mitigation efforts established by the government.

Regrading modalities of health services received by the Saudi population during the COVID-19 pandemic, the current study showed that most of the participants who experienced any health problems during the pandemic called 937 number, which was initiated by the Saudi Ministry of Health (MOH) to receive all calls from patients about health aspect, receive and monitor patients' notifications, settle them as soon as possible anytime and anywhere across Saudi Arabia, and follow them up according to international standards, provide 24/7 medical consultation through doctors, and offer key advice and guidelines for poisoning cases [18]. 

The second most used modality was having appointments through health-related applications including Seha application, Mawid application, and Asafny application (two-thirds of the participants depended on these two modalities). Primary health care visits were the third most common modality used, but this was primarily for those who were suspected to have COVID-19 infection and had to get a swab but not other health care services. The role of government hospitals was low in the listed modalities after private hospitals (where one-quarter of the participants visited to receive health care). This can be explained by the fact that government hospitals were occupied and burdened with COVID-19 cases with no capacity to deliver regular services.

Regarding participants' satisfaction with the provided health services, the study results revealed that most of the participants (more than 88%) saw that the provided services were helpful. This was reflected in their satisfaction level regarding the health services they received, where nearly the same percentage reported their satisfaction regarding these services with high satisfaction among more than half of them. Satisfaction was significantly higher among the younger participants compared to the older participants. This was mostly due to the fact that the older participants were more anxious about their health as they were one of the risky groups. As a result, any delay worried them, which affected their perception regarding the provided health care services, which could be delayed due to the pandemic. Irrespective of the limited role of the hospitals in the direct provision of health services, the highest satisfaction was reported for their attendants with good satisfaction among those who used the electronic services. Abdel Nasser A et al [19] studied patient's experience in using the telemedicine strategies during the COVID-19 pandemic and assessed these patients' perceptions about their experience of using telemedicine in Saudi Arabia. They found that about 50% of the participants were highly satisfied with the ease of registration (52%), while 43.4% of respondents reported that they could talk freely over telemedicine. The highest satisfaction was reported was 40.5% regarding the ability to understand the recommendations, 40.5% about the overall quality of care provided, 37.4% about the overall telemedicine consult experience. In Australia, Isautier JM et al [20] reported that 61.9% of the population stated that their telehealth experience was “just as good as” or “better than” their traditional in-person medical appointment experience. Generally, participants felt that telehealth would be more useful for medical appointments after the COVID-19 pandemic ends. Another study by Murphy A et al [21] revealed that 36% of participants reported medium satisfaction and 21% reported high satisfaction regarding provided health services during the COVID-19 pandemic. Access to telehealth significantly predicted overall satisfaction - 80% of the families without telehealth access reported low satisfaction compared to 30% of families with access to telehealth.

## Conclusions

In conclusion, the current study showed a high level of public satisfaction regarding different modalities of health services provided during the COVID-19 pandemic in Saudi Arabia. This satisfaction was moderately high among telehealth users and those who had hospital visits for health care. Most dissatisfied cases were due to delayed appointment times, lack of medical staff specialized, and long waiting times. The application of video consultations may help on the future of telemedicine aiming to provide healthcare for all applicants not only to chronic patients. There was no difference in satisfaction was observed based on level of education. After the reported success of this strategy, it should not be stopped when the pandemic is mitigated.
